# Two-Stage Latent Dynamics Modeling and Filtering for Characterizing Individual Walking and Running Patterns with Smartphone Sensors

**DOI:** 10.3390/s19122712

**Published:** 2019-06-17

**Authors:** Jaein Kim, Juwon Lee, Woongjin Jang, Seri Lee, Hongjoong Kim, Jooyoung Park

**Affiliations:** 1 Department of Mathematics, Korea University, 145 Anam-ro, Seongbuk-gu, Seoul 02841, Korea; kkjin85@korea.ac.kr (J.K.); hongjoong@korea.ac.kr (H.K.); 2 Department of Control and Instrumentation Engineering, Korea University, 2511 Sejong-ro, Sejong-City 30019, Korea; saero94j@korea.ac.kr (J.L.); coorung@korea.ac.kr (W.J.); tpfl27@korea.ac.kr (S.L.)

**Keywords:** latent dynamics, smartphone sensors, human movements, modeling and filtering, latent variables, machine learning applications

## Abstract

Recently, data from built-in sensors in smartphones have been readily available, and analyzing data for various types of health information from smartphone users has become a popular health care application area. Among relevant issues in the area, one of the most prominent topics is analyzing the characteristics of human movements. In this paper, we focus on characterizing the human movements of walking and running based on a novel machine learning approach. Since walking and running are human fundamental activities, analyzing their characteristics promptly and automatically during daily smartphone use is particularly valuable. In this paper, we propose a machine learning approach, referred to as ’two-stage latent dynamics modeling and filtering’ (TS-LDMF) method, where we combine a latent space modeling stage with a nonlinear filtering stage, for characterizing individual dynamic walking and running patterns by analyzing smartphone sensor data. For the task of characterizing movements, the proposed method makes use of encoding the high-dimensional sequential data from movements into random variables in a low-dimensional latent space. The use of random variables in the latent space, often called latent variables, is particularly useful, because it is capable of conveying compressed information concerning movements and efficiently handling the uncertainty originating from high-dimensional sequential observation. Our experimental results show that the proposed use of two-stage latent dynamics modeling and filtering yields promising results for characterizing individual dynamic walking and running patterns.

## 1. Introduction

Recently, data from built-in sensors such as gyroscope and accelerometers in smartphones have been readily available, and analyzing data for various types of health information from smartphone users has become a popular health care application area. Among the relevant issues in the area, one of the most prominent issues is analyzing the characteristics of human movements. In this paper, we consider the problem of characterizing the human movements of walking and running by means of a novel machine learning approach. Since many health care topics are related to walking and running, a great deal of current research efforts focus on questions and problems concerning how to characterize human walking and running patterns via machine learning. In particular, various machine learning methods have successfully addressed distinguishing human activities such as walking (see e.g., [1,2,3,4]) utilizing data from wearable sensors. Sekine et al. [1] distinguished ambulatory patterns of elderly subjects walking on stairways versus walking on level ground using waist acceleration signals, utilizing wavelet coefficients. Papagiannaki et al. [2] proposed an activity recognition scheme for older people based on feature extraction from wearable sensors and machine learning methods, and considered the problem of recognizing physical activity of older people. In their works, classification was conducted by standard machine learning as well as deep learning techniques. Jiang et al. [3] applied convolutional neural networks (CNNs) to human activity recognition using activity image, and extracted optical features for six different actions. This study [3] used the activity image, which assembled time-series sensor signals of accelerometers and gyroscopes, as input to CNNs, and obtained good performance in terms of recognition accuracy and computational cost. Wang et al. [4] proposed an algorithm for detecting several human ambulatory patterns from data obtained via a triaxial accelerometer; decomposing sensor signal data into frequency scales was conducted by a discrete Fourier transform (DFT), and then classifications of the resultant features were performed by multilayer perceptron (MLP) networks. In addition, for machine learning methods for detecting falls, one may refer to papers such as [5,6,7,8,9].

In this paper, we investigate the problem concerning how to find the low-dimensional latent dynamics for walking and running with smartphone sensors, which will lead us to some intrinsic representation of the movements. For solving this problem, we propose a machine learning approach, referred to as a ’two-stage latent dynamics modeling and filtering’ (TS-LDMF) method, where we combine a latent space modeling stage with a nonlinear filtering stage. The proposed method makes use of encoding high-dimensional sequential data from human movements into random variables in a low-dimensional latent space. The use of random variables in latent space, i.e., latent variables, is particularly useful, because this is capable of carrying compressed information concerning movements and efficiently handling uncertainty deriving from high dimensional sequential observation.

In the sense that the proposed method utilizes the step of transforming high-dimensional smartphone sensor outputs into low-dimensional latent variables, the proposed method can be viewed as fulfilling nonlinear dynamic dimension reduction. Dimension reduction is one of the most fundamental issues in the area of machine learning. Among the well-known conventional linear and nonlinear dimension reduction methods, are there principal component analysis (PCA) [10] and kernel principal component analysis (KPCA) [11]. The proposed method is a type of generalized dimension reduction, which can perform nonlinear dimension reduction with the additional capacity of modeling latent dynamics and filtering for latent states in the low-dimensional latent space.

In addition, in the sense that this paper addresses multiple functions of generating joint distributions for observations and latent variables, modeling latent dynamics, and nonlinear filtering for latent states with smartphone sensor data based on TS-LDMF, the work in this paper may be viewed as a closely related extension and applications of deep generative models such as the deep Markov model [12,13]. Deep generative models are a branch of deep learning [14,15,16], and recently, they have been successfully applied to various important classes of unsupervised learning problems such as variational auto-encoders (VAE) [17,18], generative adversarial networks (GAN) [19,20], neural ordinary differential equations (neural ODE) [21,22], and deep Markov models [12,13]. One of the main advantages of deep generative models is that uncertainty information in their probabilistic models can be explicitly provided by their solutions. Because uncertainty information in their probabilistic models is valuable in addressing latent dynamics modeling, filtering, and probability density estimation, the strategies of deep generative modeling are also useful for the purpose of this paper. For the task of finding the normal latent region for walking or running, we use a modern density estimation approach based on the neural ODE [21,22]. Since the ODE-based approach can work well utilizing a relatively smaller neural network [21,22], it will be very useful to our purpose in handling high-dimensional data on smartphones. In addition, since the latent samples yielded by the training of TS-LDMF models are utilized in our density estimation step, combining the proposed TS-LDMF together with the neural ODE [21,22]-based approach is natural and seamless. Our main contributions and novelty can be summarized as follows:

- In order to obtain low-dimensional intrinsic trajectories associated with walking and running data, we propose a novel method referred to as ’two-stage latent dynamics modeling and filtering’, which combines a latent dynamics modeling stage together with non-linear incremental filtering stage.
- The proposed method can yield simple and intrinsic representation in latent spaces for walking and running. Providing simple and intrinsic representation in latent spaces for human movements is a great help in a variety of application fields such as the entertainment, healthcare, and medical domains.
- Our works are based on smartphone data, which ensures easy accessibility and convenient deployment in real applications.

The remaining parts of this paper is organized as follows. In Section 2, after describing some common framework, we present the two-stage latent dynamic modeling and filtering method for the problem of characterizing individual dynamic walking and running patterns. In Section 3, the effectiveness of the proposed TS-LDMF method is demonstrated by experiments, and in Section 4, the usefulness of the proposed TS-LDMF method is discussed. Finally in Section 4, concluding remarks are given togather with issues for future work.

## 2. Methods

**Figure 1:**
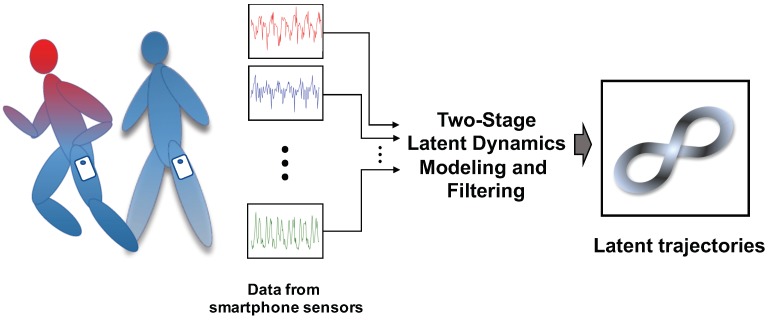
Two-stage latent dynamics modeling and filtering (TS-LDMF) for characterizing dynamic walking and running patterns in a low-dimensional latent space.

The purpose of this study is to propose a machine learning approach that can characterize low-dimensional dynamic features of individuals while walking and running with smartphone sensors. Our approach yields low-dimensional latent trajectories of human motions by processing high-dimensional raw data from smartphone sensors, as shown in Figure 1.

**Figure 2:**
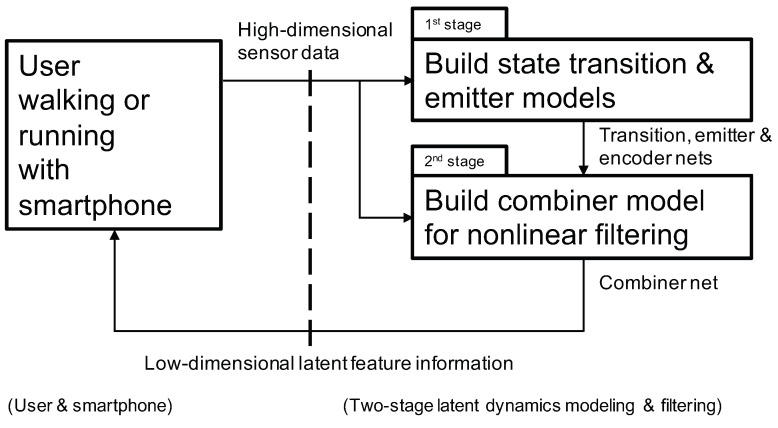
Block diagram for two-stage latent dynamics modeling and filtering (TS-LDMF) approach.

For characterizing the sequential data from movements in latent feature space, we propose a novel approach, termed a two-stage latent dynamics modeling and filtering (TS-LDMF) method. The two-stage latent dynamics modeling and filtering method combines a latent space modeling stage with a nonlinear filtering stage, for characterizing individual dynamic walking and running patterns by analyzing smartphone sensor data. The block diagram for its workflow is shown in Figure 2.

**Figure 3:**
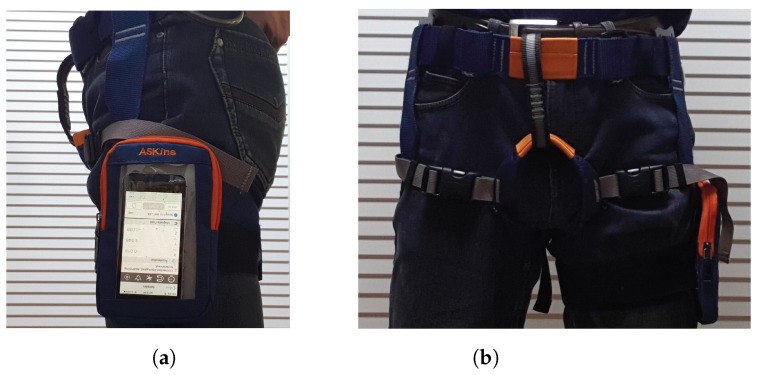
Illustrations of smartphone location and position in experiments: (**a**) side view, (**b**) front view.

An established procedure for training TS-LDMF models in experiments is provided in Table 1. In the experiment, we used the built-in sensors of the iPhone. The smartphone unit includes two types of sensors, accelerometer and gyro sensor, which can obtain motion data around three orthogonal axes (*x*, *y*, *z*). Thus, we measured motion data consisting of six features. Furthermore, for additional information on movement intensity, we consider total magnitudes [23] of acceleration and angular velocity as well. After obtaining the motion data and additional intensity features, we have an 8-dimensional feature data set, $x_{t}$, each time, *t* (Table 2). In Figure 3, we show the configuration of the smartphone unit in the experiments. In the following subsections, we describe details of the TS-LDMF method.

### 2.1. Backbone Structure of TS-LDMF

In this subsection, we describe the backbone structure of the proposed TS-LDMF, which the first and second stages of TS-LDMF utilize as a common sub-module. The backbone structure contains a transition network, an emitter network, and a probability distribution of the initial latent variable, exact meanings of which are provided in the following. In the backbone structure, we use the transition network for a process model in low-dimensional latent space, and the emitter network for a measurement model for sensors (e.g., [12,24]). For the emitter and transition networks, we use the multilayer perceptron (MLP) [25] and mixture density network [26], respectively. Under the assumption of the Markov property [12,24] in the latent dynamics, we have the following joint probability distribution for the observations, $x_{0:T}$, and the latent variables, $h_{0:T}$:

(1) $$p_{\theta }\left(x_{0:T},h_{0:T}\right)=p_{\theta }\left(h_{0}\right)p_{\theta }\left(x_{0}|h_{0}\right)\prod_{t=1}^{T}p_{\theta }\left(x_{t}|h_{t}\right)p_{\theta }\left(h_{t}|h_{t-1}\right),$$

where $p_{\theta }\left(h_{0}\right)$, $p_{\theta }\left(x_{t}|h_{t}\right)$, and $p_{\theta }\left(h_{t}|h_{t-1}\right)$ stand for the probability distribution of the initial latent variable, the conditional probability distribution for the emitter network, and the conditional probability distribution for the transition network, respectively. Note that the probabilistic model of Equation (1) is based on the key idea that the sequence of the high-dimensional sequential observation, $x_{0:T}$, can be explained by means of the lower-dimensional sequence of the latent variables, $h_{0:T}$, where the $h_{0:T}$ are generated via the conditional distribution of the transition network, $p_{\theta }\left(h_{t}|h_{t-1}\right)$, and the $x_{0:T}$ are generated via the conditional distribution of the emitter network, $p_{\theta }\left(x_{t}|h_{t}\right)$. In addition, note that in Equation (1), our notation uses θ for all the parameters of the backbone structure. When the joint distribution can be factorized as in Equation (1), the true posterior inference distribution $p_{\theta }\left(h_{0:T}|x_{0:T}\right)$ can be factorized as follows [12,27]:

(2) $$p_{\theta }\left(h_{0:T}|x_{0:T}\right)=p_{\theta }\left(h_{0}|x_{0:T}\right)\prod_{t=1}^{T}p_{\theta }\left(h_{t}|h_{t-1},x_{t:T}\right).$$

Motivated by the factored representation of Equation (2), the strategy of the variational approximation [27,28] usually approximate the true posterior distribution $p_{\theta }\left(h_{0:T}|x_{0:T}\right)$ with the variational distribution of the following form [12]:

(3) $$q_{\phi }\left(h_{0:T}|x_{0:T}\right)=q_{\phi }\left(h_{0}|x_{0:T}\right)\prod_{t=1}^{T}q_{\phi }\left(h_{t}|h_{t-1},x_{t:T}\right),$$

where ϕ stands for parameters of the variational distributions.

As mentioned, Equations (1) and (2) hold true under the assumption of the Markov property. However, since the Markov property is not guaranteed in actual experiments involving sensors, satisfying $p_{\theta }\left(x_{t},h_{t}|h_{t-1}\right)=p_{\theta }\left(x_{t}|h_{t}\right)p_{\theta }\left(h_{t}|h_{t-1}\right)$ exactly is difficult. To alleviate this difficulty, we construct the observation and latent state vectors based on the current and past two feature data sets. More specifically, our observation $y_{t}$ is defined as

(4) $$y_{t}\overset{▵}{=}\left[x_{t},x_{t-1},x_{t-2}\right],$$

where $x_{t}$ is the feature data set of Table 2 at time *t*, and the corresponding latent state vector $z_{t}$ plays the role of $\left\{h_{t},h_{t-1},h_{t-2}\right\}$. Note that as a result of this definition, there is some overlap of information among the three observation vectors, $y_{t}$, $y_{t-1}$, and $y_{t-2}$. For the distributions of the transition and emitter networks, we use normal and multinomial distributions, respectively. Based on the new observation and latent state definition, the corresponding equation involving the variational distributions becomes

(5) $$q_{\phi }\left(z_{0:T}|y_{0:T}\right)=q_{\phi }\left(z_{0}|y_{0:T}\right)\prod_{t=1}^{T}q_{\phi }\left(z_{t}|z_{t-1},y_{t:T}\right).$$

In the following Section 2.2 and Section 2.3, we explain how the true posterior distribution $p_{\theta }\left(z_{0:T}|y_{0:T}\right)$ can be adequately approximated at each stage of TS-LDMF.

### 2.2. First Stage of TS-LDMF for Modeling Latent Dynamics

The purpose of the first stage of TS-LDMF is to train the backbone structure with the variational approximation strategy. As mentioned, the true posterior distribution $p_{\theta }\left(z_{0:T}|y_{0:T}\right)$ can be efficiently approximated by the variational distribution in the form of Equation (5). For the variational distributions in the right hand side of the equation, one often uses separate normal distributions for $q_{\phi }\left(z_{0}|y_{0:T}\right)$ and $q_{\phi }\left(z_{t}|z_{t-1},y_{t:T}\right)$, i.e.,

(6) $$q_{\phi }\left(z_{0}|y_{0:T}\right)=N\left(z_{0}|\mu \left(y_{0:T}\right),\Sigma \left(y_{0:T}\right)\right),$$

(7) $$q_{\phi }\left(z_{t}|z_{t-1},y_{t:T}\right)=N\left(z_{t}|\mu \left(z_{t-1},y_{t:T}\right),\Sigma \left(z_{t-1},y_{t:T}\right)\right),t\in \left\{1,\cdots ,T\right\},$$

where N(z|μ,Σ) is the notation for the multivariate normal distribution with the mean vector μ and the covariance matrix Σ. Motivated by the observation [29] that much better training stability is obtained when the variational distribution $q_{\phi }$ for $z_{t}$ depends exclusively on the data $y_{t:T}$, we use the modification for $q_{\phi }\left(z_{t}|z_{t-1},y_{t:T}\right)$ in the first stage so that it be conditioned exclusively on $y_{t:T}$, and modeling interactions with $z_{t-1}$ work only through the transition network. Note that under this modification, both $q_{\phi }\left(z_{0}|y_{0:T}\right)$ and $q_{\phi }\left(z_{t}|z_{t-1},y_{t:T}\right)$ can be implemented with the common form:

(8) $$q_{\phi }\left(z_{t}|y_{t:T}\right),t\in \left\{0,\cdots ,T\right\}.$$

More specifically, our implementation for $q_{\phi }\left(z_{t}|y_{t:T}\right)$ uses the hidden state $h_{t}^{rnn}$ of a recurrent neural network structure running backwards in time across $y_{t:T}$, and for the recurrent neural network structure, we use the gated recurrent units (GRU) [30]. We call the network used for $q_{\phi }$ in the first stage the encoder network. In the training process of the first stage, we find the parameters θ and ϕ simultaneously by maximizing ELBO(θ,ϕ), the variational lower bound of Equation (9) [17,18]:

(9) $$\log p\left(y_{0:T}\right)\geq ELBO\left(\theta ,\phi \right)=E_{z_{0:T}∼q_{\phi }\left(z_{0:T}|y_{0:T}\right)}\left[\log p_{\theta }\left(y_{0:T}|z_{0:T}\right)\right]-KL\left(q_{\phi }\left(z_{0:T}|y_{0:T}\right)\|p_{\theta }\left(z_{0:T}\right)\right).$$

More precisely, ELBO(θ,ϕ) of Equation (9) can be written as follows:

(10) $$\sum_{t=0}^{T}E_{z_{t}∼q_{\phi }\left(z_{t}|y_{t:T}\right)}\left[\log p_{\theta }\left(y_{t}|z_{t}\right)\right]-KL\left(q_{\phi }\left(z_{0}|y_{0:T}\right)\|p_{\theta }\left(z_{0}\right)\right)-\sum_{t=1}^{T}E_{z_{t-1}∼q_{\phi }\left(z_{t-1}|y_{t-1:T}\right)}[KL\left(q_{\phi }\left(z_{t}|y_{t:T}\right)\|p_{\theta }\left(z_{t}|z_{t-1}\right)\right].$$

**Figure 4:**
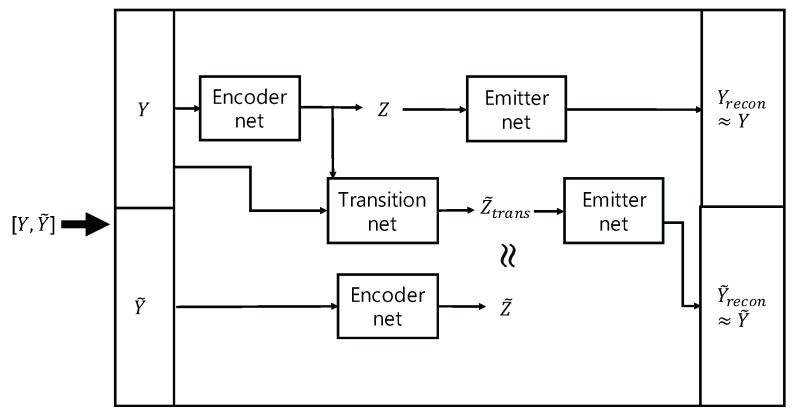
Interpretation for training the first stage of TS-LDMF.

By denoting $y_{0:T-1}$ and $y_{1:T}$ as *Y* and $\tilde{Y}$, respectively, one can see that the training of the first stage can be interpreted as aiming at the following goals (Figure 4):

(11) $$Y_{recon}\approx Y,{\tilde{Y}}_{recon}\approx \tilde{Y},\mathrm{and}{\tilde{Z}}_{trans}\approx \tilde{Z},$$

where $Z\overset{▵}{=}z_{0:T-1},\tilde{Z}\overset{▵}{=}z_{1:T}$, and ${\tilde{Z}}_{trans}$ is the random variables produced by means of the transition network. In Equation (11), both $Y_{recon}\approx Y\mathrm{and}{\tilde{Y}}_{recon}\approx \tilde{Y}$ mean that reconstructions generated by the emitter network should be close to actual observations so that the log-likelihood of observations be large, while ${\tilde{Z}}_{trans}\approx \tilde{Z}$ means that distributions of latent variables yielded by the encoder network and the transition networks should be close in the sense of Kullback-Leibler divergence [31]. Note that the interpretation shown in Figure 4 is quite general, and may be applicable in other types of variational approaches as well.

### 2.3. Second Stage of TS-LDMF for Estimating Latent Variables

**Figure 5:**
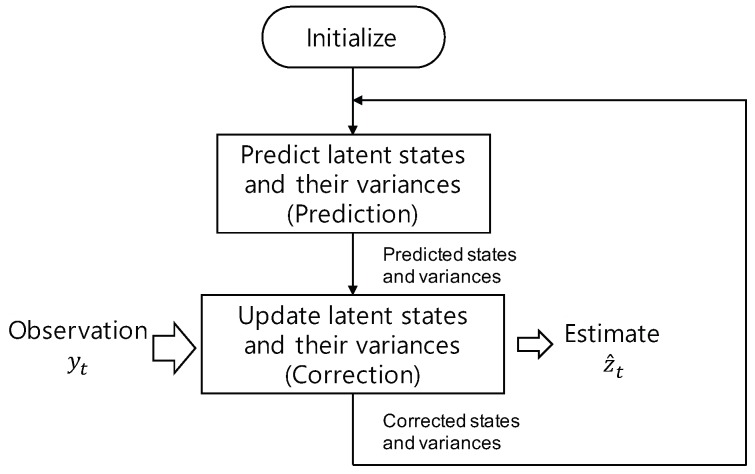
Flowchart for the filtering performed by the second stage of TS-LDMF.

The proposed TS-LDMF consists of two stages, i.e., the first stage for latent dynamics modeling, and the second stage for estimating latent variables via nonlinear filtering. As indicated in Figure 2, training the first stage of TS-LDMF yields a transition network, an emitter network, and an encoder network, which are for $p_{\theta }\left(z_{t}|z_{t-1}\right)$, $p_{\theta }\left(y_{t}|z_{t}\right)$, and $q_{\phi }\left(z_{t}|y_{t:T}\right)$ each time step *t*, respectively. Note that in the encoder network, the computation of the conditional distribution for $z_{t}$ involves the future sequence of observation, $y_{t:T}$; hence the encoder network obtained by training the first stage has a limitation in estimating latent states with sequential data processing. The goal of TS-LDMF is to provide a versatile way for characterizing individual dynamic walking and running patterns so that it can work sequentially and efficiently when characterizing, estimating, and predicting the latent trajectories for movements. With this type of versatility in mind, we introduce the second stage of TS-LDMF for estimating latent variables via sequential data processing. In the second stage, we use a new variational distribution $q_{\psi }$, which is different from the $q_{\phi }$ of the first stage, and does not rely on the future sequence of observation. We call the resultant network for $q_{\psi }$ the combiner network, and for its implementation, we use the multilayer perceptron [25]. Our implementation of the combiner network for $z_{t}$ uses the predicted state, predicted variance, and the observation $y_{t}$ as its inputs, which is motivated by the way that the state and covariance are updated in the correction step of linear and extended Kalman filters [32,33]. A flowchart for the filtering performed by the second stage of TS-LDMF is shown in Figure 5. Note that in the training process of the second stage, we optimize the parameters of the combiner network only, and the transition and emitter networks for $p_{\theta }\left(z_{t}|z_{t-1}\right)$ and $p_{\theta }\left(y_{t}|z_{t}\right)$ remain fixed as provided by the first stage.

## 3. Experimental Results

In our experiments, we addressed the problem of characterizing individual human motions with smartphone sensors via the proposed two-stage latent dynamics modeling and filtering method. For these motions, we considered walking and running. We believe that the proposed method can be applicable to more types of motions, and we are planning to study its applicability in future continuing research.

### 3.1. Data Collection

Based on the procedure of Table 1, the experiments were conducted at the Korea University R&D Center with its WiFi networks. In experiments for the paper, we recruited 10 male and 10 female subjects to evaluate the performance of the proposed method properly. Profiles of the recruited subjects are given in Table 3.

During the entire experimental procedure, we used a single smartphone unit: the iPhone SE, one laptop computer (a MacBook Pro), and two applications: Matlab [34] and PyTorch [35] for processing sensor data, and training the TS-LDMF models, respectively. The walking and running data were sent from the smartphone to the computer over the campus WiFi network. Figure 3 shows the configuration of the smartphone in the experiments.

We set the sampling rate for data transmission from the smartphone to the PC at 30 Hz. Training was conducted with sensor data from the smartphone accelerometer and gyro sensors after minmax scaling. The minmax scaling was conducted by importing the MinMaxScaler from sklearn.preprocessing [36]. Note that the scaling part is somewhat user-dependent, because its min and max values should be chosen so that the resultant interval should cover all the subjects’ data. As shown in Figure 3, the smartphone near the left pants pocket, positioned on the side of the leg with a harness and with the screen facing outward. In the experiment, the participants walked and ran a predefined course. We thereby acquired the necessary data for simulation of the proposed method. A detailed protocol for obtaining the data for each subject is as follows:

(a) Set the predefined course for walking and running.
(b) Set parameter (sampling rate: 30 Hz, data collection time: 60 s) with help of MATLAB Support Package for Apple iOS Sensors.
(c) Run Matlab Mobile on the iPhone.
(d) Connect the iPhone to the desktop on the same Wifi network.
(e) Position the iPhone to the predefined location and position.
(f) Instruct the subject to walk on the predefined course.
(g) Initiate the countdown prior to the data recording.
(h) Let the subject begin walking before completing the countdown.
(i) Upon completion of the data recording, have the subject stop.
(j) Save the recorded data (angular velocity around the x, y, z-direction; acceleration along the x, y, z-direction) and conduct preprocessing for data (total magnitude of angular velocity and total magnitude of acceleration) on the desktop.
(k) Repeat steps (d) through (j) for running.

In addition, a detailed description of the unit’s feature data set is provided in Table 2. Note that each feature data set for the configuration consists of eight dimensions. For the latent space, we chose $R^{2}$ or $R^{3}$ for convenience of visualization and easy understanding.

### 3.2. Experimental Results

This section explains the experimental environment and data for demonstrating the latent space based solutions for characterizing dynamic walking and running patterns.

For demonstrating the latent space based solutions, we used five sets of training data. In all these training data, a sequence of features was obtained from our twenty subjects with a frequency of 30 Hz. Additionally, the training batch size was 64. In all experiments, we constructed the observation of Equation (4) from the current and past two feature data sets; hence, the dimension of the resultant observation each time is 8×3=24. Note that some overlap of information exists among the three consecutive observation vectors. Since the sampling rate in this paper is set at 30 Hz, there is no loss of information even with the overlapping information compared to our previous related work [9], where the sampling rate was set at 10 Hz and no overlapping information was allowed.

For splitting data into train and test sets, we used the five-fold cross-validation. More precisely, we utilized the corresponding method of the sklearn library [36], i.e., sklearn.model_selection.KFold(n_splits=5). Note that the method provides train and test indices to split data into train and test sets, and splits the data into k=5 consecutive folds. Based on the method, each fold was used as a test set whereas the remaining k−1=4 folds constituted the training set. Thus, 20% of data was used as a test set while the remaining 80% of data was used for training.

**Figure 6:**
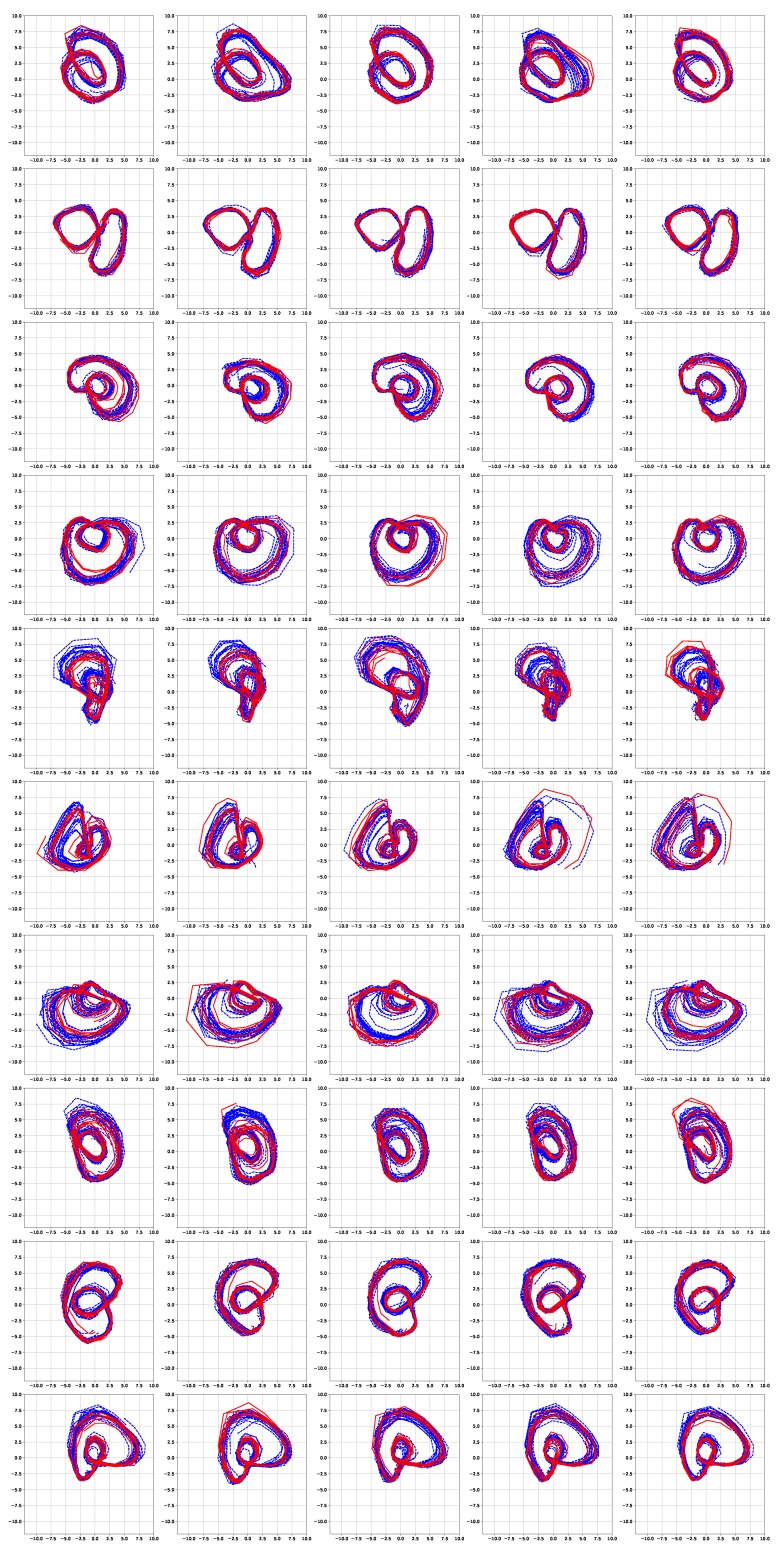
Five-fold cross validation results: Latent trajectories in $R^{2}$ for walking of 10 male participants. Solid red lines and dashed blue lines for test data and training data, respectively.

**Figure 7:**
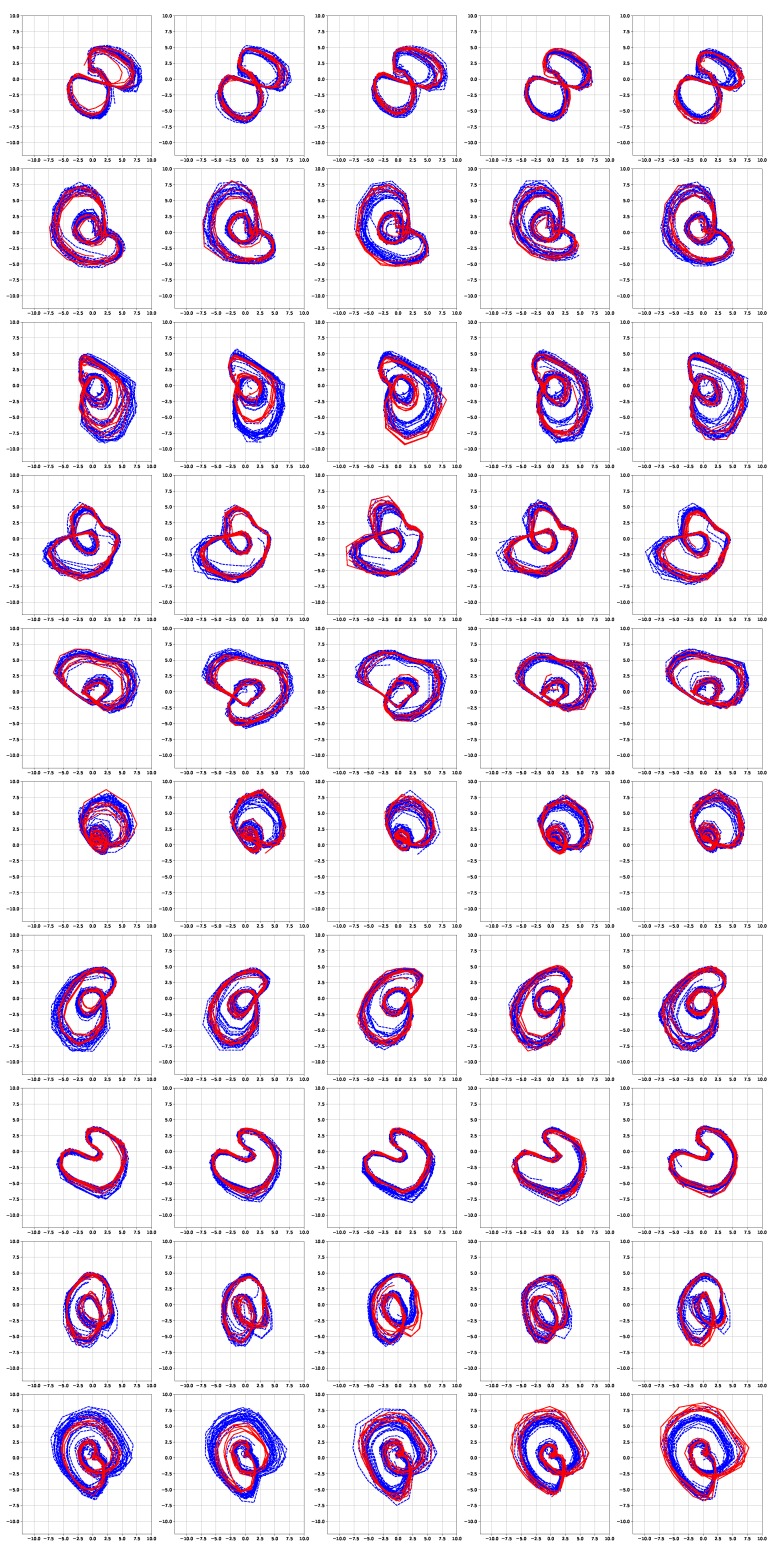
Five-fold cross validation results: Latent trajectories in $R^{2}$ for walking of 10 female participants. Solid red lines and dashed blue lines for test data and training data, respectively.

**Figure 8:**
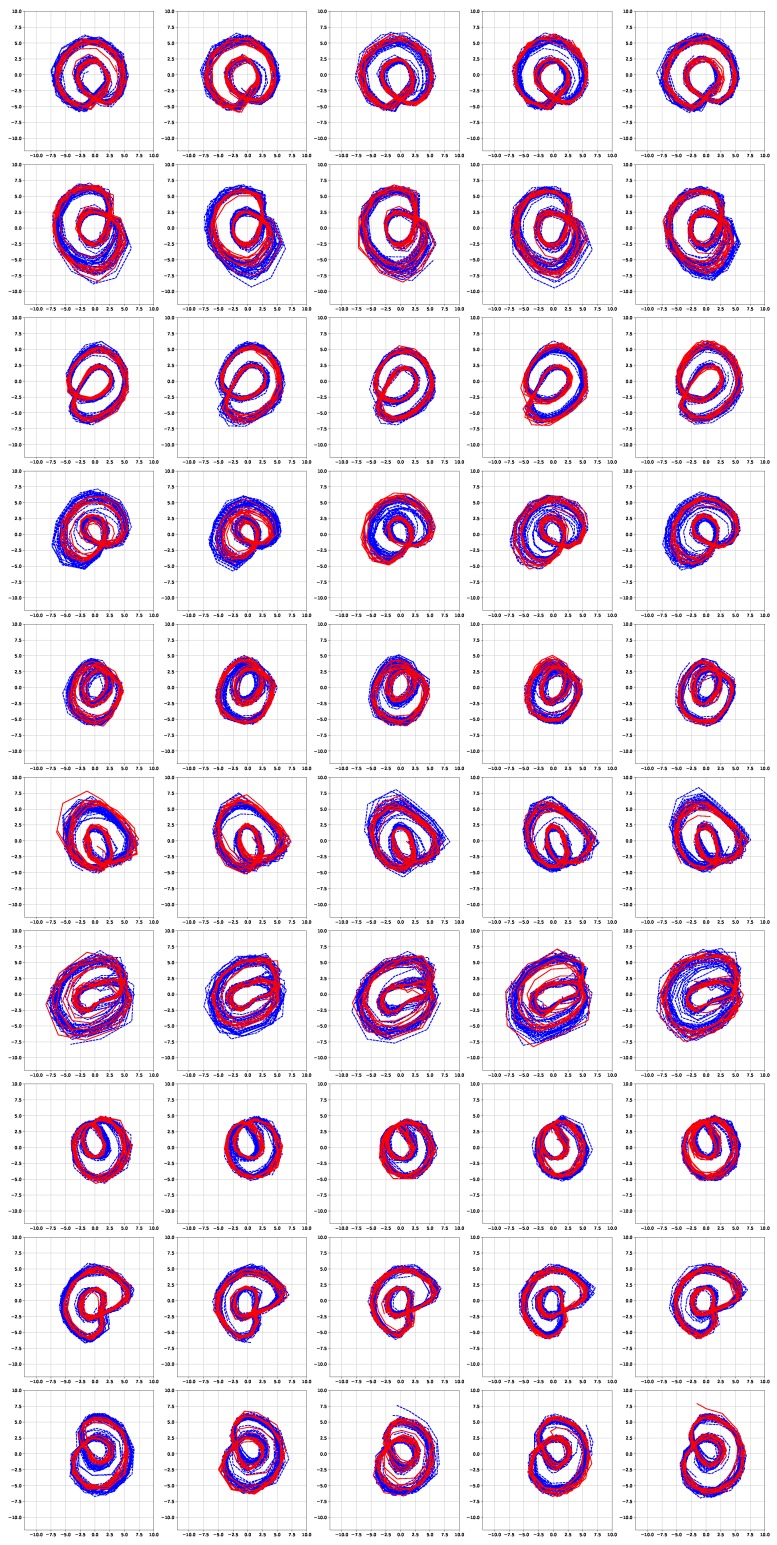
Five-fold cross validation results: Latent trajectories in $R^{2}$ for running of 10 male participants. Solid red lines and dashed blue lines for test data and training data, respectively.

**Figure 9:**
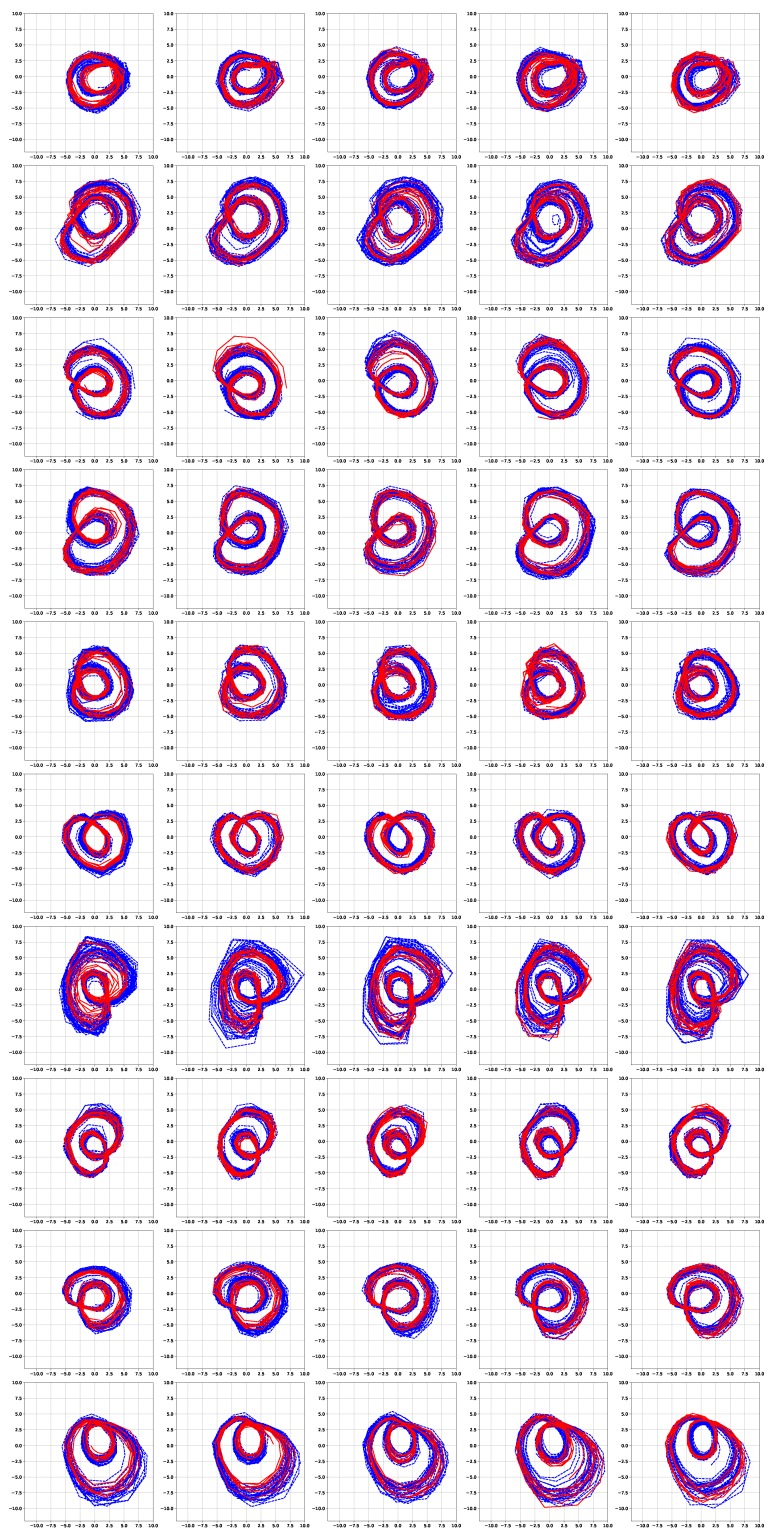
Five-fold cross validation results: Latent trajectories in $R^{2}$ for running of 10 female participants. Solid red lines and dashed blue lines for test data and training data, respectively.

**Figure 10:**
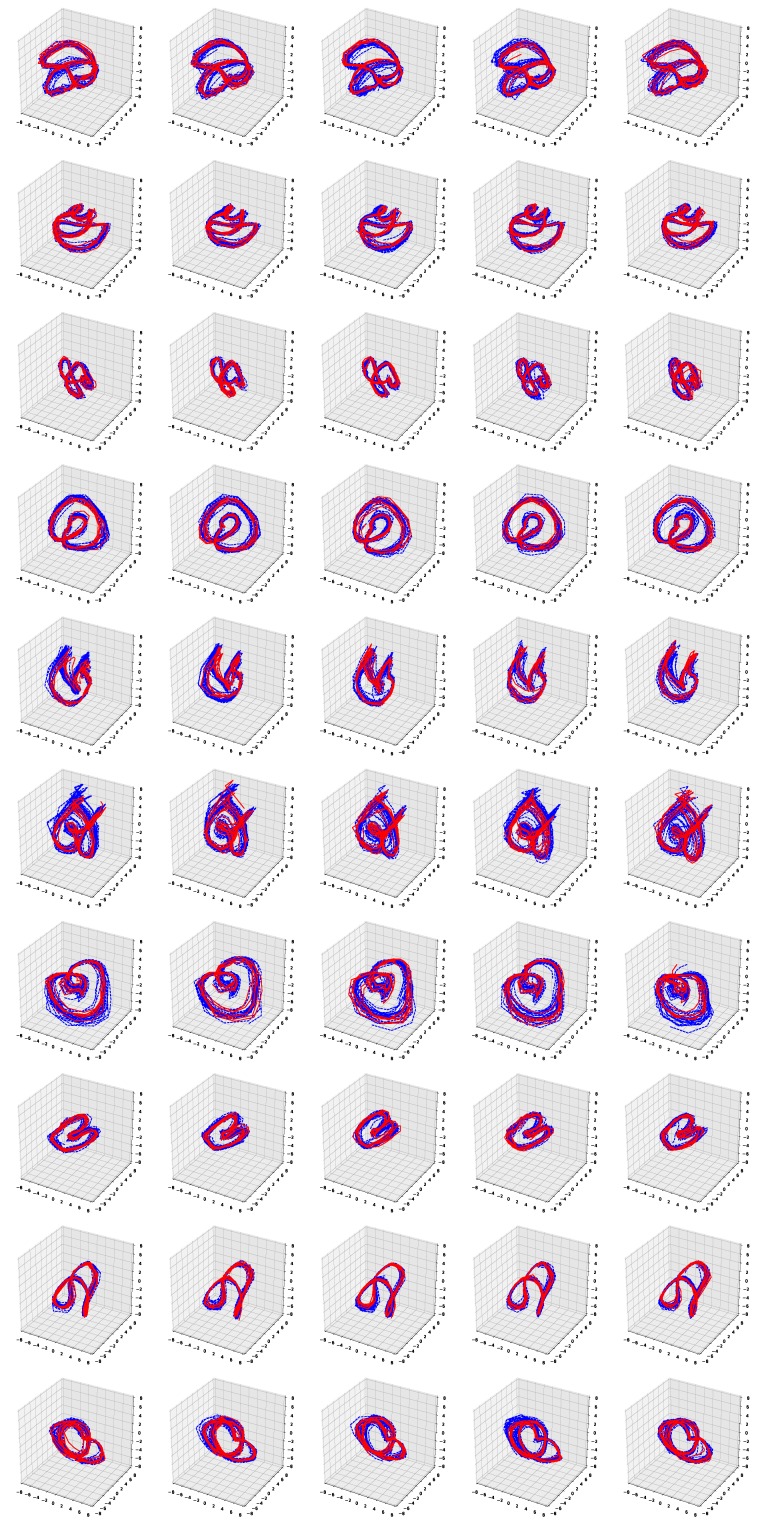
Five-fold cross validation results: Latent trajectories in $R^{3}$ for walking of 10 male participants. Solid red lines and dashed blue lines for test data and training data, respectively.

**Figure 11:**
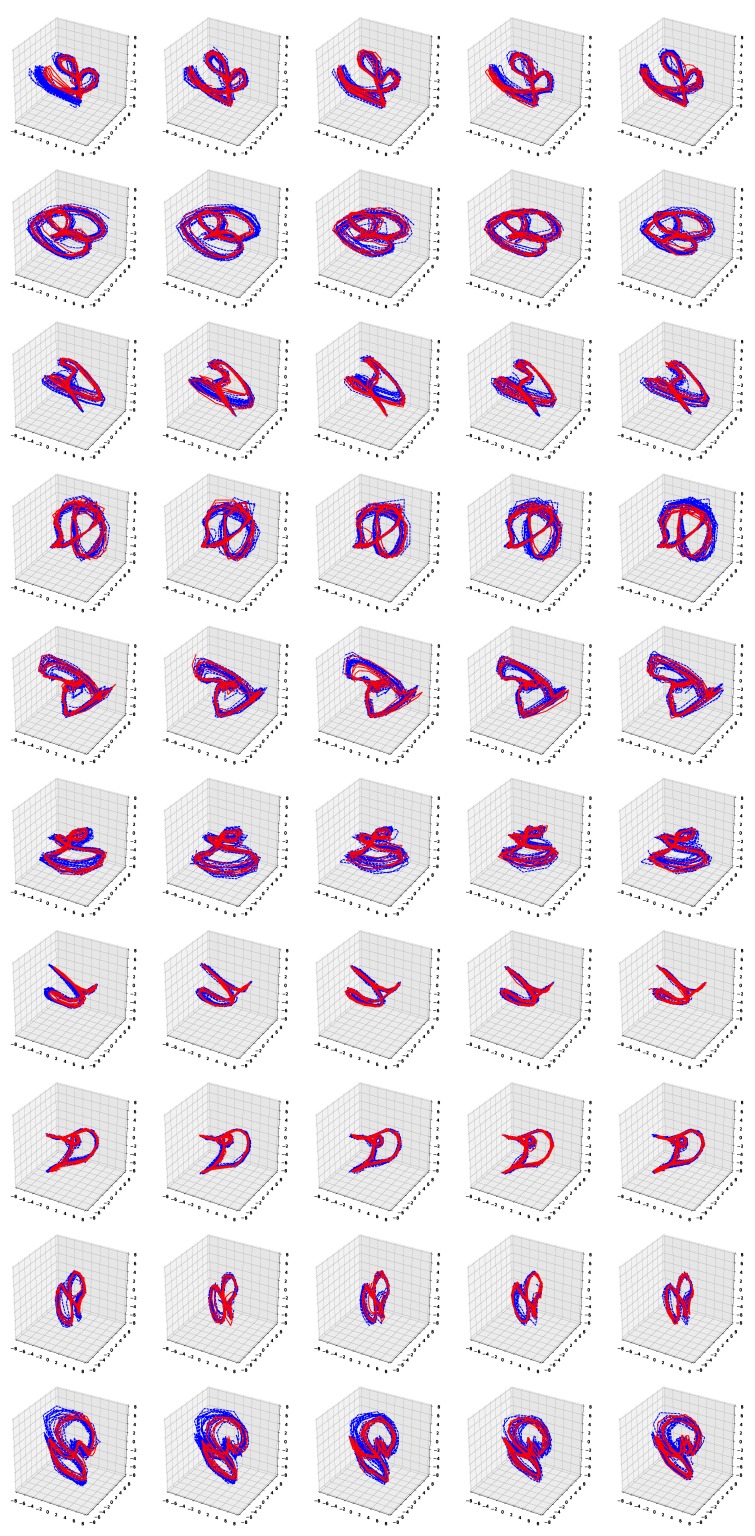
Five-fold cross validation results: Latent trajectories in $R^{3}$ for walking of 10 female participants. Solid red lines and dashed blue lines for test data and training data, respectively.

**Figure 12:**
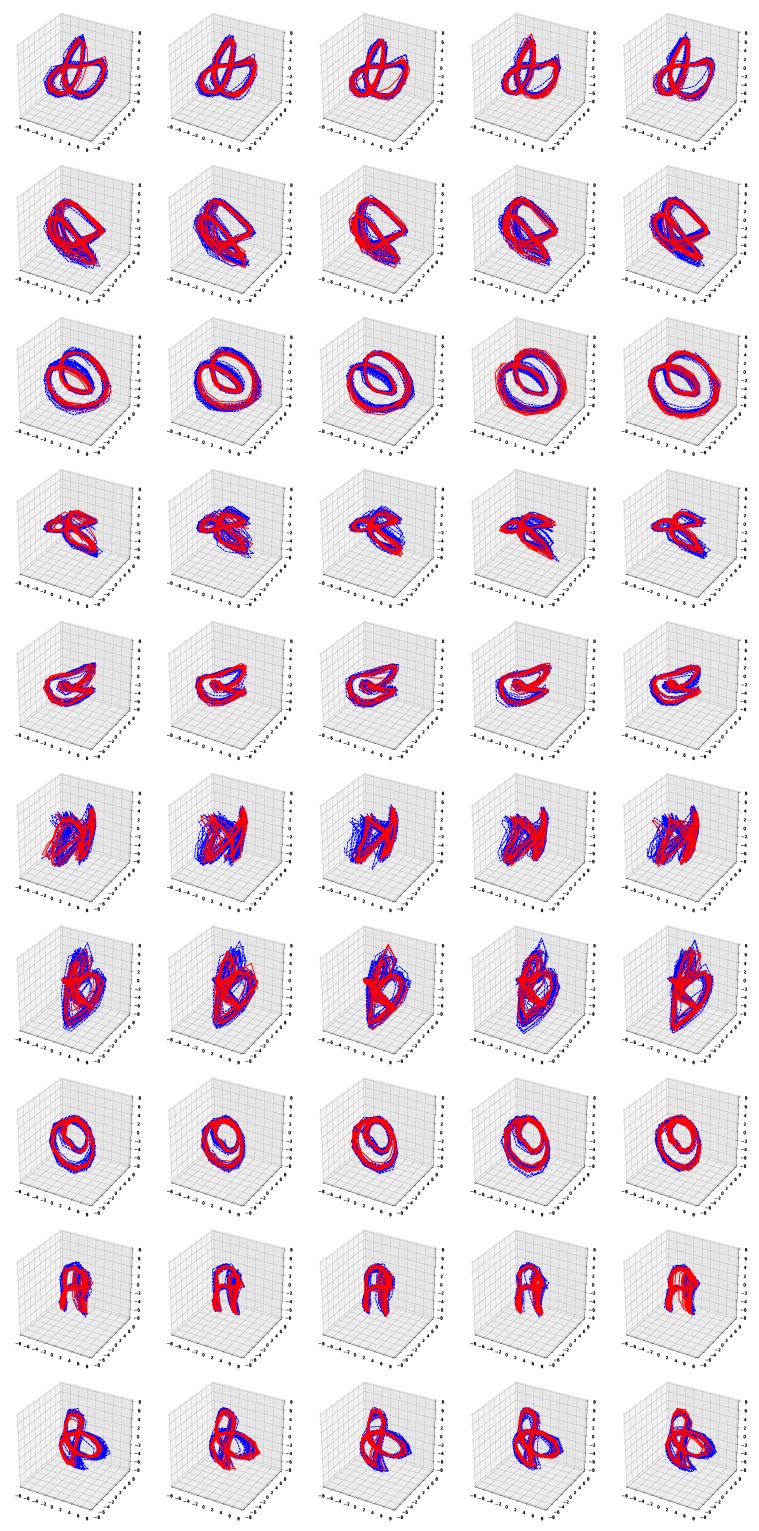
Five-fold cross validation results: Latent trajectories in $R^{3}$ for running of 10 male participants. Solid red lines and dashed blue lines for test data and training data, respectively.

**Figure 13:**
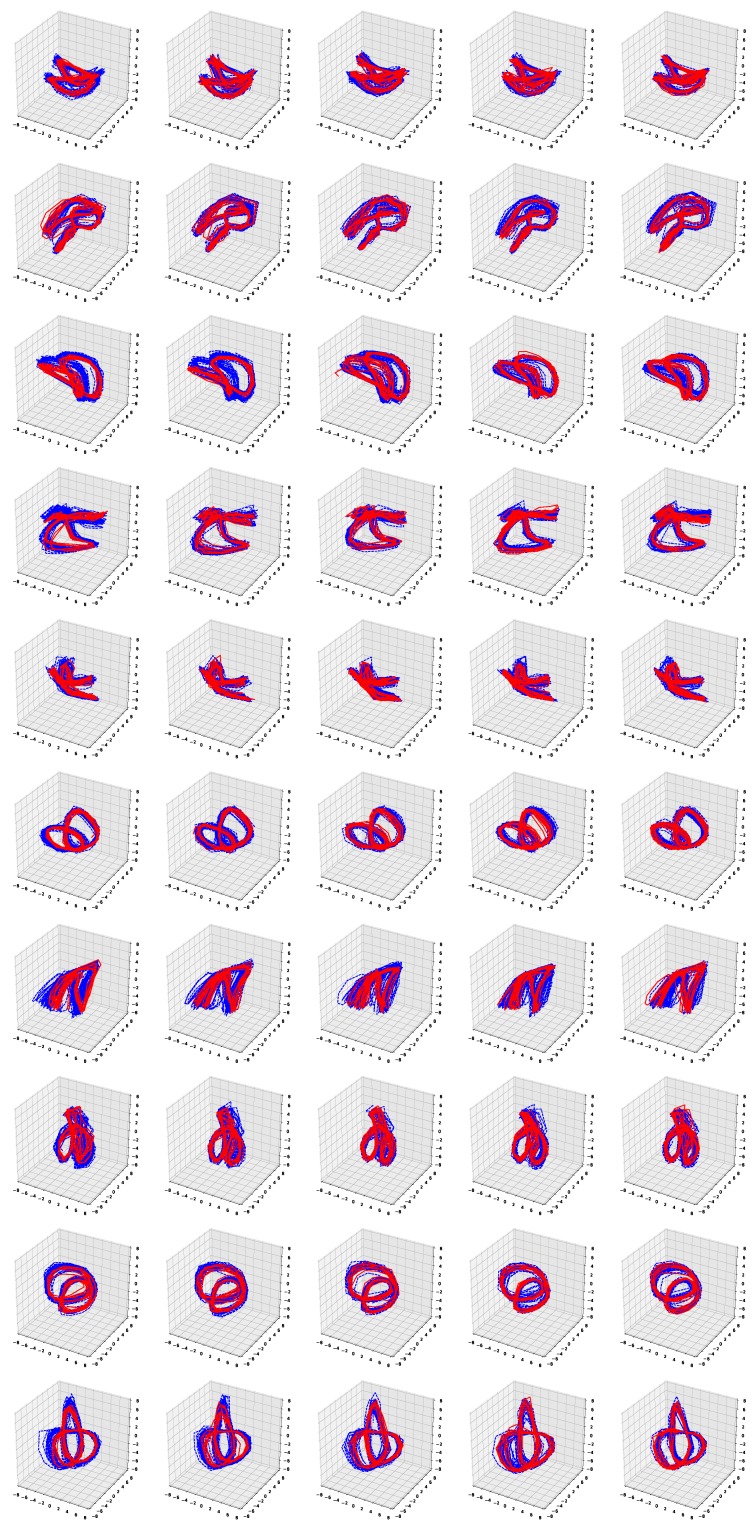
Five-fold cross validation results: Latent trajectories in $R^{3}$ for running of 10 female participants. Solid red lines and dashed blue lines for test data and training data, respectively.

Figure 6 and Figure 7 show the simulation results of the five-fold cross-validation for characterizing individual dynamic walking patterns with the proposed TS-LDMF method, and show the resulting latent trajectories in $R^{2}$ for male and female subjects, respectively. Note that in each cross-validation, we considered the setup that the training and test data include only data from one person. The subplots in the figure can be interpreted as follows: in the *j*-th row, which is for the *j*-th subject, the *i*-th subplot shows the latent trajectories obtained from the proposed TS-LDMF method for the *i*-th experiment, in which the *i*-th walking data set was used as the test set, and the other four walking data sets were used as the training set for estimating latent trajectories. In each individual subplot, the solid red line indicates a portion of the latent trajectories from the test data sets provided by the proposed method, while the dashed blue lines represents some portion of the latent trajectories of the training data sets. Figure 6 and Figure 7 indicate that the proposed latent space based method worked satisfactorily in characterizing dynamic walking patterns in the latent space. From the cross-validation results, obvious similarities can be seen between the latent trajectory of the test data and that of the training data. For the purpose of characterizing individual dynamic running patterns, we also conducted similar experiments. Figure 8 and Figure 9 show the results of the corresponding five-fold cross-validation for male and female subjects, respectively. As shown in the Figures, the proposed latent space based method worked well for characterizing the dynamic running patterns, and the cross-validation results of Figure 8 and Figure 9 exhibit obvious similarities between the latent trajectories of the training data and those of the test data. In addition, Figure 10, Figure 11, Figure 12 and Figure 13 show the corresponding results for the case with the three dimensional latent space.

Overall, the results of Figure 6, Figure 7, Figure 8, Figure 9, Figure 10, Figure 11, Figure 12 and Figure 13 indicate that the proposed method successfully transformed high-dimensional sequences of noisy observation data from the smartphone sensors to low-dimensional latent trajectories, and the training and test data with their common characteristics in fact shared similar patterns in latent space.

### 3.3. Performance Comparison

For performance comparison with a conventional approach, we considered incremental principal component analysis [37]. The family of principal component analysis methods such as PCA (principal component analysis), PPCA (probabilistic principal component analysis), and IPCA (incremental principal component analysis) are all important tools for reducing dimensionality, and have often been utilized for problems involving gait and dimension reduction (e.g., [38]).

We compared the results of the proposed method to those of the incremental PCA-based method using the mean squared distance (MSE), which is defined as

(12) $$MSE\overset{▵}{=}\frac{1}{M}\sum_{k=1}^{M}{\left\|x_{k}-{\hat{x}}_{k}\right\|}^{2}$$

where *M* is the number of test patterns, $x_{k}$ is the *k*th test pattern, and ${\hat{x}}_{k}$ is the reconstructed result for the *k*th test pattern. Table 4 shows the ratios of $MSE_{IPCA}/MSE_{TS-LDMF}$ computed for the test set of the five-fold cross-validation. Ratios being larger than one in the table shows that the proposed method performs better in terms of reconstruction capability, compared to the incremental principal component analysis.

**Figure 14:**
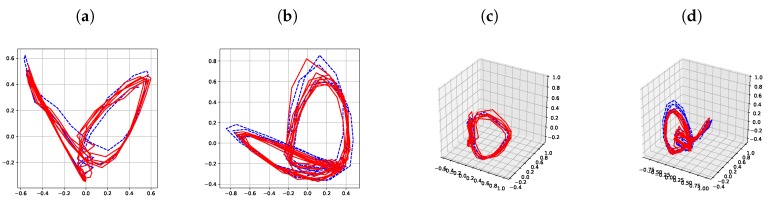
Latent trajectories obtained by the incremental principal component analysis method for the first cross-validation set of the first subject: (**a**) 2-dim latent trajectories for walking, (**b**) 2-dim latent trajectories for running, (**c**) 3-dim latent trajectories for walking, (**d**) 3-dim latent trajectories for running. Solid red lines and dashed blue lines for test data and training data, respectively.

Figure 14 shows latent trajectories obtained by the incremental principal component analysis for the first cross-validation case of the first subject. The corresponding results of Figure 6, Figure 7, Figure 8, Figure 9, Figure 10, Figure 11, Figure 12 and Figure 13 indicate that our results are more smooth and easy to interpret, compared to the IPCA results of Figure 14.

**Figure 15:**
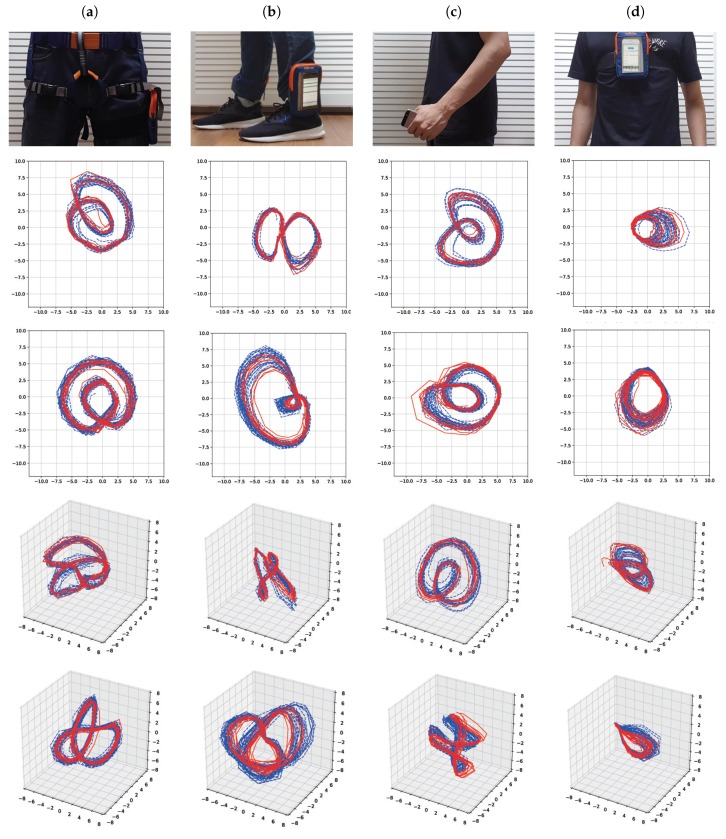
Location of smartphone together with the latent trajectories in $R^{2}$ and $R^{3}$ for walking and running for the first cross-validation of the first subject: (**a**) Thigh, (**b**) foot, (**c**) hand, (**d**) chest. Solid red lines and dashed blue lines for test data and training data, respectively.

Finally in order to show that the effectiveness of the proposed method does not depend on particular locations of smartphone, we also considered other choice of locations used in related works [39,40,41,42] (Figure 15). Each column of Figure 15 shows the location of smartphone together with the latent trajectories of walking and running for the first cross-validation set of the first subject. From the figure, one can see that regardless of locations, the proposed method can yield reasonable intrinsic latent trajectories for walking and running. Additionally, from the figure, one can conclude that when the location of the smartphone has more movements (e.g., foot or hand), the resultant latent trajectories tend to have more variations compared to the case with less movements (e.g., chest).

## 4. Discussion and Conclusions

### 4.1. Discussion

In this paper, we investigated the use of machine learning for characterizing dynamic walking and running patterns with smartphone sensors. The key idea behind the characterizing is that the sequence of the high-dimensional observation can be explained by means of the substantially lower-dimensional sequence of the latent variables. We believe that the key idea is reasonable because the high-dimensional observation in our experiments all originates from human motions, which are intrinsically movements in a three dimensional space. For the task of characterizing dynamic walking and running patterns in a low-dimensional latent feature space, we put forth a novel approach, referred to as two-stage latent dynamics modeling and filtering. Our approach is closely related with the deep Markov model (DMM) approach [12]. The most important difference worth noting is that the proposed method uses the second stage to estimate latent variables via filtering in the training phase. The second stage is critically important, because it ensures that the resultant networks can work in real time. Since the latent trajectories obtained by the proposed TS-LDMF method are somewhat unique for each subject, the proposed TS-LDMF method has the potential value of identifying individual dynamic walking and running patterns with smartphone sensor data. In our opinion, the capability of TS-LDMF for characterizing individual dynamic walking and running patterns can successfully be extended to other types of human motions. Furthermore, we believe that despite specific experimental environment of these experiments for verifying the proposed method, this method can be deployed in existing smartphone systems.

**Figure 16:**
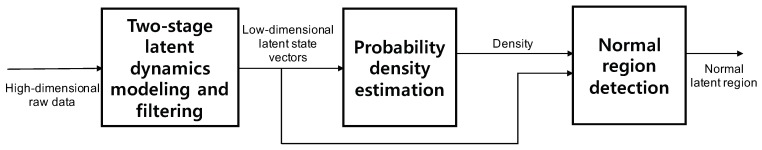
Pipeline graph for the combination of TS-LDMF and density estimation for normal latent region of walking or running.

**Figure 17:**
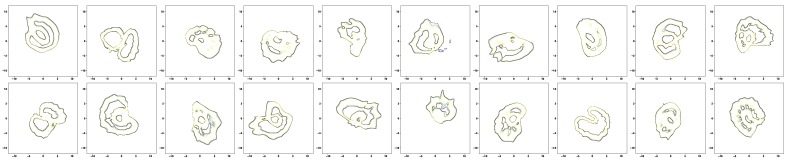
Density estimation results in $R^{2}$ for walking of 20 subjects.

**Figure 18:**
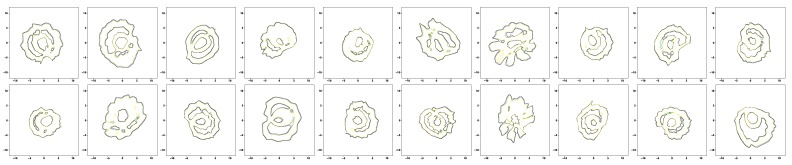
Density estimation results in $R^{2}$ for running of 20 subjects.

Once training the TS-LDMF models is completed, we can find the normal latent regions for movements based on the training results. For the task of finding the normal latent region for walking or running, we use a modern density estimation approach based on the neural ODE [21,22] in obtaining a probability density for latent samples resulting from training the TS-LDMF models. This ODE-based method [21,22] is particularly attractive because it can parameterize the derivative of the latent state by means of a relatively smaller neural network. Additionally, since the latent samples generated by the training of the two-stage latent dynamics modeling and filtering can be recycled in our density estimation step, these two modules can be combined together seamlessly. The pipeline graph for the combination is shown in Figure 16. Given any observation data, the two-stage latent dynamics modeling and filtering is capable of providing a probability density for their corresponding latent patterns. Consequently, one can obtain the support of the latent objects by thresholding the resultant probability density function. Figure 17 and Figure 18 show how relevant contours for the density of latent patterns in $R^{2}$ appeared in the experiments. For these contours for the density, we utilized matplotlib.pyplot.contour [43]. Since it can be quickly noticed if the trajectory deviates from the high-density normal region, the combination may be utilized to anomaly detection problems such as fall detection.

### 4.2. Conclusions

In this study, we have examined the problem of characterizing individual dynamic walking and running patterns with smartphone sensors. For the sensors, we used built-in sensors in a single smartphone unit positioned near the left pants pocket, and from this unit, acceleration, rate of turn along three perpendicular axes, and their total magnitudes were used as input features.

For characterizing movements, we proposed two-stage latent dynamics modeling and filtering, which can map noisy high-dimensional sequential observation to low-dimensional latent trajectory, and the resultant latent trajectories efficiently found intrinsic characteristics of the users’ dynamic walking and running patterns. The latent trajectories found by the proposed method showed that the low-dimensional latent trajectories associated with dynamic walking and running patterns were smooth, while the original input features from smartphone sensors were often noisy.

For the task of finding the normal latent region for walking or running, we used a modern density estimation approach based on the neural ODE, and utilized latent samples resulting from the two-stage latent dynamics modeling and filtering. Since the latent samples generated from training for two-stage latent dynamics modeling and filtering can be recycled in the task, combining the TS-LDMF and the ODE-based density estimation can be done seamlessly. Future work to be done includes further, more extensive experiments and comparison studies, which might uncover strengths and weaknesses of the proposed approach, as well as further refinements of the proposed method in various directions and with more participants. Examination of different types of neural networks and applications to other kinds of human motions are some of the topics to be covered along these lines. Issues of deploying trained networks into current smartphone systems so that they work in real-time are also reserved for future studies. 

## Figures and Tables

**Table 1:** An established procedure for training TS-LDMF models in experiments.

1: Obtain the training data for each category of motions (walking or running), and for each subject.
2: Obtain the test data for each category of motions (walking or running), and for each subject.
3: Train the first stage of TS-LDMF, and fix its transition and emitter networks after the training is completed.
4: Train the second stage of TS-LDMF, and fix its combiner network after the training is completed.
5: Find latent trajectories corresponding to the training and test data for each category of motions (walking or running), and for each subject.
6: Validity check: If the the obtained latent trajectories are not satisfactory, repeat the the above steps until satisfactory.
7: Report TS-LDMF results (i.e., the transition, emitter, and combiner networks), and the latent trajectories for each class of motions (walking or running) and for each subject.

**Table 2:** Smartphone unit’s feature data set.

Notation	Meaning
$\omega_{x},\omega_{y},\omega_{z}$	Angular velocities around the x,y,z-directions, respectively
$\omega_{T}$	Square root of the sum of squares of angular velocities, $\sqrt{\omega_{x}^{2}+\omega_{y}^{2}+\omega_{z}^{2}}$
$A_{x},A_{y},A_{z}$	Accelerations along the x,y,z-directions, respectively
$A_{T}$	Square root of the sum of squares of accelerations, $\sqrt{A_{x}^{2}+A_{y}^{2}+A_{z}^{2}}$

**Table 3:** Profiles of the recruited subjects.

Subjects	Gender	Age (yrs)	Height (cm)	Weight (kg)
subject 1	Male	35	174	62
subject 2	Male	25	175	80
subject 3	Male	26	167	56
subject 4	Male	28	185	84
subject 5	Male	58	172	64
subject 6	Male	37	170	70
subject 7	Male	49	165	85
subject 8	Male	28	181	100
subject 9	Male	31	170	80
subject 10	Male	59	172	67
subject 11	Female	29	163	58
subject 12	Female	47	167	58
subject 13	Female	56	158	63
subject 14	Female	36	153	47
subject 15	Female	23	163	55
subject 16	Female	22	160	48
subject 17	Female	21	159	54
subject 18	Female	21	165	48
subject 19	Female	24	163	68
subject 20	Female	22	161	52
Average	–	33.85	167.15	64.95

**Table 4:** Comparison of average mean squared distance (MSE) ratios, $MSE_{IPCA}/MSE_{TS-LDMF}$, (ICPA = incremental principal component analysis) after reconstruction.

	2-dim, walk	2-dim, run	3-dim, walk	3-dim, run
Male subjects	1.01	1.75	1.21	1.60
Female subjects	1.33	1.69	1.36	1.51
Average	1.17	1.72	1.29	1.56
